# Mercury Exposure and Antinuclear Antibodies among Females of Reproductive Age in the United States: NHANES

**DOI:** 10.1289/ehp.1408751

**Published:** 2015-02-10

**Authors:** Emily C. Somers, Martha A. Ganser, Jeffrey S. Warren, Niladri Basu, Lu Wang, Suzanna M. Zick, Sung Kyun Park

**Affiliations:** 1 Department of Internal Medicine,; 2 Department of Environmental Health Sciences,; 3 Department of Obstetrics and Gynecology, and; 4 Division of Clinical Pathology, Department of Pathology, University of Michigan, Ann Arbor, Michigan, USA; 5 Faculty of Agricultural and Environmental Sciences, McGill University, Montreal, Quebec, Canada; 6 Department of Biostatistics,; 7 Department of Family Medicine, and; 8 Department of Epidemiology, University of Michigan, Ann Arbor, Michigan, USA

## Abstract

**Background:**

Immune dysregulation associated with mercury has been suggested, although data in the general population are lacking. Chronic exposure to low levels of methylmercury (organic) and inorganic mercury is common, such as through fish consumption and dental amalgams.

**Objective:**

We examined associations between mercury biomarkers and antinuclear antibody (ANA) positivity and titer strength.

**Methods:**

Among females 16–49 years of age (*n* = 1,352) from the National Health and Nutrition Examination Survey (NHANES) 1999–2004, we examined cross-sectional associations between mercury and ANAs (indirect immunofluorescence; cutoff ≥ 1:80). Three biomarkers of mercury exposure were used: hair (available 1999–2000) and total blood (1999–2004) predominantly represented methylmercury, and urine (1999–2002) represented inorganic mercury. Survey statistics were used. Multivariable modeling adjusted for several covariates, including age and omega-3 fatty acids.

**Results:**

Sixteen percent of females were ANA positive; 96% of ANA positives had a nuclear speckled staining pattern. Geometric mean (geometric SD) mercury concentrations were 0.22 (0.03) ppm in hair, 0.92 (0.05) μg/L blood, and 0.62 (0.04) μg/L urine. Hair and blood, but not urinary, mercury were associated with ANA positivity (sample sizes 452, 1,352, and 804, respectively), after adjusting for confounders: for hair, odds ratio (OR) = 4.10 (95% CI: 1.66, 10.13); for blood, OR = 2.32 (95% CI: 1.07, 5.03) comparing highest versus lowest quantiles. Magnitudes of association were strongest for high-titer (≥ 1:1,280) ANA: hair, OR = 11.41 (95% CI: 1.60, 81.23); blood, OR = 5.93 (95% CI: 1.57, 22.47).

**Conclusions:**

Methylmercury, at low levels generally considered safe, was associated with subclinical autoimmunity among reproductive-age females. Autoantibodies may predate clinical disease by years; thus, methylmercury exposure may be relevant to future autoimmune disease risk.

**Citation:**

Somers EC, Ganser MA, Warren JS, Basu N, Wang L, Zick SM, Park SK. 2015. Mercury exposure and antinuclear antibodies among females of reproductive age in the United States: NHANES. Environ Health Perspect 123:792–798; http://dx.doi.org/10.1289/ehp.1408751

## Introduction

Autoimmune disorders, although individually rare, are collectively estimated to afflict 7.6–9.4% of Americans (Cooper et al. 2009) and are among the 10 leading causes of death among women (Thomas et al. 2010; Walsh and Rau 2000). Almost all autoimmune diseases have a strong preponderance among females, with female to male ratios of up to 9:1 and onset often occurring during mid-adulthood (Cooper and Stroehla 2003; Somers et al. 2007, 2014).

Autoimmunity, which can include autoantibody formation, represents a breakdown of tolerance against self-antigens (Lleo et al. 2010). Self-reactive lymphocytes may occur in healthy individuals, and in the absence of related pathology, autoimmunity represents pre- or subclinical immune dysregulation. Thus, the term “autoimmunity” should be distinguished from autoimmune disease, because it does not denote clinical or symptomatic disease. Data are sparse regarding the prognostic significance of preclinical autoimmunity or the “conversion” rate to particular disorders, although autoantibodies may precede autoimmune diagnoses by several years (Arbuckle et al. 2003) and nearly all autoimmune diseases are characterized by circulating autoantibodies (Scofield 2004). Antinuclear antibodies (ANAs) are highly sensitive for a variety of autoimmune conditions, including systemic lupus erythematosus (SLE), scleroderma, and Sjögren’s syndrome. Estimates of ANA prevalence in individuals without autoimmune disease vary widely (1–24%) (Fritzler et al. 1985; Rosenberg et al. 1999) due to differing methodologies and population characteristics. ANA prevalence of approximately 13% has been reported in key studies using a 1:80 titer cutoff (Satoh et al. 2012; Tan et al. 1997) based on an immunofluorescence assay, the method recommended by the American College of Rheumatology as the gold standard for ANA testing (Meroni and Schur 2010).

Mercury is a ubiquitous and persistent toxicant with pleiotropic effects, and it is currently ranked as a top three priority pollutant by the U.S. Agency for Toxic Substances and Disease Registry (2011). Consumption of seafood, particularly of large species, is a common source of organic mercury (methylmercury) exposure (Mergler et al. 2007). It has been estimated that in the United States each year, approximately 8% of mothers and 0.6 million newborns have mercury concentrations exceeding levels considered by regulatory agencies to be safe (Trasande et al. 2005). Immunotoxic effects, including autoantibody production, have been clearly demonstrated in murine models in response to both organic and inorganic mercury (Pollard et al. 2010). In humans, occupational mercury exposure (predominantly inorganic and elemental species) among miners has been associated with increased risk of autoimmunity (Gardner et al. 2010; Silva et al. 2004), and an increased risk of SLE has been reported among dental professionals (Cooper et al. 2004). However, immune effects associated with low levels of exposure to each type of mercury in the general population are not well characterized (Mergler et al. 2007).

Because the biologic effects, sources, and patterns of exposure to organic and inorganic mercury are expected to differ, it is important to examine both species. Biomarkers of mercury exposure in humans include hair mercury, representing predominantly organic (methyl) mercury; total blood mercury, a combination of organic and inorganic mercury; and urinary mercury, a marker predominantly of inorganic/elemental mercury. In the U.S. National Health and Nutrition Examination Survey (NHANES), hair mercury was measured in adult females (16–49 years of age) but not in males.

Using NHANES data, we explored the associations between three types of biomarkers of mercury exposure and the presence, strength, and patterns of ANAs in a representative sample of reproductive-age females from the U.S. population.

## Methods

*Study population*. NHANES is conducted by the Centers for Disease Control and Prevention (CDC), National Center for Health Statistics (NCHS) (CDC/NCHS 2015b). It uses a stratified, multistage probability cluster design, with oversampling of selected subpopulations, to obtain a representative sample of the civilian, noninstitutionalized U.S. population. NHANES protocols were approved by the NCHS Institutional Review Board, and informed consent was obtained. In our study we used data from three cycles of continuous NHANES data (1999–2004) (CDC/NCHS 2015a). Participation rates were 76% for 1999–2000, 80% for 2001–2002, and 76% for 2003–2004 (CDC/NCHS 2013). The eligible population for our analysis included female participants 16–49 years of age who completed a physical examination with biospecimen collection for ANA and mercury assessment.

From a total of 5,984 females 16–49 years of age in NHANES 1999–2004, 1,932 were included in the one-third subsample with ANA assessment, of whom 1,354 had available ANA data. Hair mercury was available for one cycle (1999–2000), total blood mercury for three (1999–2004), and urinary mercury for two (1999–2002). For hair, blood, and urinary mercury, respectively, samples sizes were 452, 1,352, and 804 (after excluding 16, 2, and 29 persons with missing data).

*ANAs*. As detailed elsewhere (CDC/NCHS 2012), standard methodology for ANA screening was used, involving indirect immunofluorescence with HEp-2 substrate for detection of IgG antibodies to cellular antigens. Titers to which fluorescence remained positive (serial dilution range, 1:80–1:1,280) and staining patterns were determined for positive specimens. ANA patterns refer to indirect immunofluorescence patterns (e.g., speckled, nucleolar, homogeneous) reflecting the anatomic distribution of intracellular antigens, and thus different nuclear components. A variety of different ANAs can give rise to a given pattern. Follow-up immunoprecipitation was used for identification of specific antigens from a standard panel.

*Mercury exposure assessment*. Three types of biomarkers for mercury exposure were used: hair (organic), total blood (organic and inorganic), and urine (predominantly inorganic/elemental). One-centimeter hair segments were utilized (approximating exposure during the preceding 2.5 months). Standard methodology for mercury measurement was used, as described elsewhere (CDC/NCHS 2005, 2007). In brief, for hair, cold vapor atomic fluorescence spectrometry following analyte extraction was used. For blood and urine, flow-injection cold vapor atomic absorption spectrometry (PerkinElmer Flow Injection Mercury System-400) was used in NHANES 1999–2002, and inductively coupled plasma mass spectrometry (ICP-MS; PerkinElmer ELAN 6100) was used in 2003–2004. Limits of detection (LODs) for hair mercury varied by batch and ranged between 0.011 and 0.027 ppm (method detection limit); 6% of the females in our study had hair mercury levels below the LOD. LODs for total blood mercury varied according to cycle and batch, ranging between 0.14 and 0.2 μg/L. Of the females in our study, 7.4%, 6.5%, and 7.5% had total blood mercury levels below the LOD for the three cycles, respectively. We did not separately investigate the inorganic fraction of blood mercury because of the large proportion < LOD (97.4%, 95.1%, and 77.2% for the three cycles). The LOD for urinary mercury was 0.14 μg/L, with 13.3% and 14.3% of the participants having urinary mercury levels < LOD for the two cycles, respectively.

*Other variables*. Sociodemographic data were collected by self-administered questionnaires. Body mass index (BMI), calculated as kilograms of body weight divided by height in meters squared, was included due to the role of obesity in chronic inflammation. Serum cotinine (nanograms per milliliter), a marker of active and passive smoking, was measured by isotope dilution–high-performance liquid chromatography/atmospheric pressure chemical ionization tandem mass spectrometry; tobacco exposure has been linked to increased risk of autoimmune diseases (Costenbader and Karlson 2006). C-reactive protein (CRP), a nonspecific inflammatory marker, was quantified (milligrams per deciliter) by latex-enhanced nephelometry. Nutrient data were estimated based on a multiple pass, computer-assisted dietary interview of food and beverage consumption, with recall assessment of individual foods consumed in the previous 24 hr. We derived data on selenium (micrograms), eicosapentaenoic acid (20:5 n-3), and docosahexaenoic acid (22:6 n-3), all found in seafood; omega-3 fatty acid intake was calculated as eicosapentaenoic acid plus docosahexaenoic acid. Selenium potentially mitigates effects of mercury (Cuvin-Aralar and Furness 1991), and omega-3s have anti-inflammatory effects (Simopoulos 2002). Among participants who underwent the dietary interview, weekly seafood intake was estimated based on recall of fish/shellfish consumption in the previous 30 days. Serum polychlorinated biphenyls (PCBs) were measured by high-resolution gas chromatography/isotope dilution high-resolution mass spectrometry (CDC 2006). A summary measure for coplanar (dioxin-like) polychlorinated biphenyls (cPCBs), which included congeners with suspected immunotoxicity (Wolff et al. 1997), was calculated as the sum of the products of the concentration of each serum lipid–adjusted congener (PCBs 81, 105, 118, 126, 156, 157, 167, 169) and its corresponding 2005 World Health Organization-defined toxic equivalency factor (TEF) (Van den Berg et al. 2006). An alternate PCB summary measure was the sum of the lipid-adjusted values for the four most prevalent PCB congeners (118, 138, 153, 180) (Laden et al. 2010); three of these are noncoplanar and without defined TEFs to take into account. To address the potential for drug-induced autoimmunity, we assessed use within the past month of four prescription medications that have been implicated in this phenomenon (procainamide, hydralazine, carbamazepine, and minocycline) (Schoonen et al. 2010).

*Statistical analysis*. To account for the complex, stratified, and multistage cluster sampling design, analyses were conducted using the survey packages of Stata (v.12; StataCorp) and R (v.2.11.1; R Foundation) to obtain appropriate estimates and standard errors. ANAs were measured in a one-third subsample, and we constructed and applied weights to our subsample according to NCHS analytic guidelines (Johnson et al. 2013). Values below the LOD for laboratory assays were handled as the LOD divided by the square root of 2. Hair and blood mercury were log-transformed due to their skewed nature, or handled as quantiles based on distributions in the study population. Two-sample *t*-tests for survey data and the Rao-Scott chi-square test were used for continuous and categorical data, respectively. *p*-Values < 0.05 were considered significant. Crude models included mercury as the independent variable; separate models were performed for each source of mercury (hair, blood, urine). Multivariable logistic regression was utilized to estimate adjusted odds ratios (ORs) for ANA positivity in association with mercury exposure. Model A included age, race/ethnicity, education, serum cotinine, and selenium; an indicator for the NHANES cycle was included in models combining data across cycles to account for potential methodological differences between cycles. Models B and C were further adjusted for omega-3 fatty acids and seafood intake, respectively, which have been suggested to negatively confound health effects of mercury (Budtz-Jørgensen et al. 2007; Guallar et al. 2002). Multivariable urinary mercury models adjusted for urinary creatinine to account for dilution of spot urine specimens. We performed sensitivity analyses adding BMI and CRP in Models A–C as potential confounders for all mercury types. Separate sensitivity analyses were performed, including the coplanar and prevalent PCB measures. For urinary mercury, we also conducted models excluding persons with impaired renal function [glomerular filtration rate (GFR) < 60 mL/min/1.73 m^2^] to account for potential reverse causation whereby chronic kidney disease may increase urinary mercury excretion. Piecewise continuous models were constructed, and linearity with the log-odds of ANA was examined by predicted probability plots with natural cubic splines with four degrees of freedom (three for hair). Multinomial logistic regression was utilized to examine ANA titer strength as the outcome, with negative ANA (< 1:80) as the base outcome, and low/moderate titer (1:80–1:640) and high titer (≥ 1:1,280) as the other outcome levels.

## Results

*Participant characteristics*. Characteristics of the study population, according to ANA positivity, are summarized in Table 1. Sociodemographics were largely similar for ANA-positive and -negative persons, although in the first cycle there was a larger proportion of non-Hispanic whites and Mexican Americans among ANA positives (*p* = 0.08). In the combined three-cycle population, education level differed between ANA-positive and -negative groups, with a higher proportion of ANA-positive adults having less than a high school education (*p* = 0.04). Among ANA-positive compared with negative participants, hair and total blood mercury levels in cycle 1 were higher (*p* = 0.03 and *p* = 0.06, respectively), and blood mercury in the three-cycle population was nonsignificantly higher.

**Table 1:** Participant characteristics according to antinuclear antibody (ANA) positivity among females 16–49 years of age in the general U.S. population (NHANES).

Characteristic	NHANES 1999–2000			NHANES 1999–2004		
	ANA positive^*a*^(*n *= 56)	ANA negative^*a*^(*n *= 396)	*p*‑Value	ANA positive^*a*^(*n *= 213)	ANA negative^*a*^(*n *= 1,139)	*p*‑Value
Age (years)	31.7 ± 2.8	32.7 ± 0.7	0.71	33.7 ± 0.02	33.6 ± 0.06	0.14
Race/ethnicity			0.08			0.25
White, non-Hispanic	15 (74.2)	135 (64.1)		85 (71.5)	458 (64.4)
Black, non-Hispanic	12 (10.7)	73 (10.7)		48 (12.9)	256 (12.9)
Mexican American	26 (12.3)	136 (7.3)		63 (8.7)	306 (9.0)
Other Hispanic	2 (2.6)	32 (9.9)		13 (5.1)	65 (6.5)
Others	1 (0.2)	20 (8.1)		4 (1.8)	54 (7.1)
Education			0.28			0.04
< High school	9 (18.1)	74 (14.8)		48 (17.2)	227 (14.7)
High school graduate/GED	15 (34.3)	61 (20.2)		38 (21.9)	198 (22.1)
Some college/graduate school	16 (43.3)	134 (52.7)		88 (55.8)	446 (55.1)
Youth (16–19 years)	16 (4.3)	127 (12.3)		39 (5.1)	268 (8.2)
BMI (kg/m^2^)	26.6 ± 1.1	27.3 ± 0.5	0.57	26.6 ± 0.1	27.5 ± 0.2	0.11
Smoking^*b*^			0.77			0.32
Never	27 (60.7)	171 (57.1)		111 (61.2)	529 (55.6)	
Former	7 (17.4)	40 (15.0)		28 (15.1)	130 (15.3)	
Current	5 (21.9)	58 (28.0)		34 (23.8)	212 (29.2)	
Serum cotinine (ng/mL)^*c*^	0.68 ± 0.43	0.75 ± 0.25	0.87	0.32 ± 0.11	0.62 ± 0.11	0.07
C-reactive protein (mg/dL)^*c*^	0.13 ± 0.03	0.18 ± 0.02	0.30	0.14 ± 0.00	0.18 ± 0.02	0.25
< 1 mg/dL	51 (84.5)	336 (89.3)	0.54	193 (92.1)	978 (87.4)	0.57
≥ 1 mg/dL	5 (15.6)	60 (10.7)		20 (7.9)	161 (12.6)	
Selenium, dietary (μg)^*c*^	87.9 ± 9.2	78.7 ± 4.0	0.30	85.5 ± 2.3	82.8 ± 1.6	0.52
Omega-3, dietary (g)^*c*^	0.02 ± 0.01	0.03 ± 0.003	0.57	0.02 ± 0.003	0.02 ± 0.001	0.57
Hair Hg (ppm)^*c*^	0.27 ± 0.04	0.21 ± 0.03	0.03	NA	NA	NA
Total blood Hg (μg/L)^*c*^	1.31 ± 0.17	1.01 ± 0.12	0.06	0.97 ± 0.00	0.91 ± 0.05	0.43
Urinary Hg (μg/L)^*c*^	0.80 ± 0.16	0.65 ± 0.08	0.34	0.67 ± 0.03^*d*^	0.62 ± 0.05^*d*^	0.56
ANA titer			NA			NA
Negative (≤ 1:40)	NA	396 (100)		NA	1,139 (100)
1:80	0	NA		0	NA
1:160	2 (1.2)	NA		3 (0.4)	NA
1:320	16 (21.3)	NA		42 (17.6)	NA
1:640	14 (18.1)	NA		64 (32.8)	NA
≥ 1:1,280	24 (59.5)	NA		104 (49.2)	NA
ANA pattern^*e*^			NA			NA
Nuclear (all)	52 (98.9)	NA		208 (99.7)	NA
Speckled	50 (94.4)	NA		203 (96.3)	NA
Nucleolar	4 (3.1)	NA		14 (5.1)	NA
Homogeneous	1 (4.4)	NA		3 (2.3)	NA
Abbreviations: Hg, mercury; NA, not applicable. ^***a***^Values are mean ± SD or *n* (%); means and percentages are weighted. ^***b***^Smoking data were available for ages ≥ 20 years (*n *= 308 for first cycle; *n *= 1,044 for 3 cycles). ^***c***^Geometric mean ± geometric SD. ^***d***^Data for NHANES 1999–2002 (*n *= 804; data were unavailable for 2003–2004). ^***e***^Three major types of nuclear staining patterns were tabulated, and more than one ANA pattern is possible; 3.1% (cycle 1) and 5.1% (cycles 1–3) of participants had more than one pattern.						

*ANAs and mercury*. The weighted proportion of participants with ANA positivity was 12% for cycle 1 and 16% for cycles 1–3. Among ANA positives, the speckled pattern was predominant (> 94%; Table 1). The geometric means (geometric SDs) for mercury corresponding to all participants included in Table 1 for whom data were available were as follows: hair, 0.22 (0.03) ppm; total blood, 0.92 (0.05) μg/L; urine, 0.62 (0.04) μg/L. Correlations between the sources of mercury in cycle 1 were as follows: hair and total blood (*r* = 0.69; *p* < 0.01), hair and urine (*r* = 0.34; *p* < 0.01), total blood and urine (*r* = 0.41; *p* < 0.01). Among the females in this study, 12.8% had a total blood mercury level > 3.5 μg/L, the reference dose extrapolated from the U.S. Environmental Protection Agency cord blood mercury reference dose of 5.8 μg/L (Mahaffey et al. 2004; Stern and Smith 2003).

Based on multivariable logistic regression (Table 2), we detected positive and statistically significant associations [confidence intervals (CIs) > 1] between ANA positivity and hair and total blood mercury, but not urinary mercury. From the multivariable models incorporating omega-3 fatty acids (Model B), the adjusted OR for ANA positivity comparing females in the highest versus lowest tertile of hair mercury was 4.10 (95% CI: 1.66, 10.13), and for the highest versus lowest quartile of blood mercury was 2.32 (95% CI: 1.07, 5.03).

**Table 2:** Association between mercury (Hg) exposure and antinuclear antibody (ANA) positivity among females 16–49 years of age in the general U.S. population (NHANES).

Hg exposure	ANA positive*n* (%)^*a*^	Crude model^*b*^OR (95% CI)	Model A^*c*^OR (95% CI)	Model B^*d*^OR (95% CI)	Model C^*e*^OR (95% CI)
Hair Hg (ppm)^*f*^
Tertile 1 (< 0.11)	14 (8)	1.00 (referent)	1.00 (referent)	1.00 (referent)	1.00 (referent)
Tertile 2 (0.11–0.27)	22 (12)	1.58 (0.27, 9.33)	2.45 (0.47, 12.82)	2.70 (0.57, 12.80)	2.28 (0.40, 12.94)
Tertile 3 (0.271–5.96)	20 (14)	1.83 (0.54, 6.16)	4.01 (1.57, 10.28)	4.10 (1.66, 10.13)	3.75 (1.06, 13.28)
Total blood Hg (μg/L)^*g*^
Quartile 1 (< 0.4)	30 (10)	1.00 (referent)	1.00 (referent)	1.00 (referent)	1.00 (referent)
Quartile 2 (0.4–0.8)	71 (19)	2.18 (1.05, 4.52)	2.25 (1.08, 4.68)	2.25 (1.09, 4.66)	2.27 (1.06, 4.83)
Quartile 3 (0.9–1.5)	51 (16)	1.72 (0.82, 3.59)	2.04 (0.94, 4.46)	2.03 (0.95, 4.33)	2.14 (0.89, 5.12)
Quartile 4 (1.6–32.8)	61 (17)	1.84 (0.88, 3.87)	2.33 (1.05, 5.19)	2.32 (1.07, 5.03)	2.51 (1.04, 6.03)
Urinary Hg (μg/L)^*h,i*^
Quartile 1 (< 0.0029)	28 (12)	1.00 (referent)	1.00 (referent)	1.00 (referent)	1.00 (referent)
Quartile 2 (0.0029–0.0063)	30 (10)	0.73 (0.35, 1.53)	0.88 (0.42, 1.87)	0.88 (0.40, 1.94)	0.89 (0.42, 1.88)
Quartile 3 (0.0063–0.0135)	28 (17)	1.30 (0.55, 3.09)	1.36 (0.58, 3.20)	1.36 (0.58, 3.20)	1.40 (0.60, 3.25)
Quartile 4 (0.0137–0.8873)	33 (12)	0.90 (0.41, 1.96)	1.18 (0.49, 2.82)	1.18 (0.49, 2.83)	1.20 (0.50, 2.90)
^***a***^Weighted percent. ^***b***^Crude models included Hg as the independent variable; separate models were performed for each source of Hg (hair, blood, urine). ^***c***^Adjusted for age, race/ethnicity, education, serum cotinine, selenium, and indicator for NHANES cycle for multicycle models. ^***d***^Model A + further adjusted for omega-3 fatty acids. ^***e***^Model A + further adjusted for total seafood intake. ^***f***^NHANES 1999–2000, one cycle (*n *= 452). ^***g***^NHANES 1999–2004, three cycles (*n *= 1,352). ^***h***^NHANES 1999–2002, two cycles (*n *= 804). ^***i***^Included all participants with urinary Hg data, irrespective of availability of hair or blood data; all urinary Hg models (including crude) adjusted for urinary creatinine.					

We performed sensitivity analyses including BMI and CRP as covariates in the logistic regression models for all mercury types and found no substantive changes in results. Likewise, in separate sensitivity analyses incorporating each PCB summary measure, the mercury associations with ANA positivity remained similar (see Supplemental Material, Table S1). Of the eight study participants (0.78 weighted percent) reporting utilization of a prescription drug associated with drug-induced autoimmunity (four using carbamazepine, four using minocycline), none were ANA positive. In the urinary mercury sensitivity analyses excluding participants with GFR < 60 mL/min/1.73 m^2^, there were no substantive changes in results.

**Figure 1:**
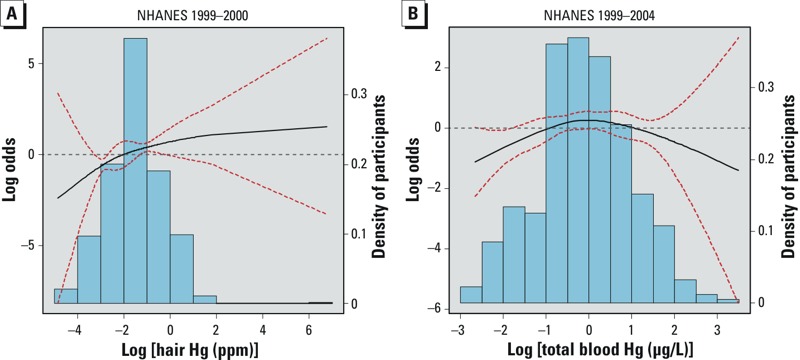
Associations of antinuclear antibody (ANA) positivity with log-transformed hair and total blood mercury (Hg), adjusted for Model B covariates. (*A*) Hair Hg (1999–2000; *n *= 452). (*B*) Total blood Hg (1999–2004; *n *= 1,352). Solid black lines represent the smoothing trends estimated from the natural spline with 3 degrees of freedom (df) for hair Hg and 4 df for total blood Hg (knots at 25th, 50th, and 75th percentiles); red dotted lines represent 95% CIs; and bars represent the weighted density distribution for Hg. The dose–response relationship for both hair and total blood Hg increased in a statistically significant fashion within the lower ranges of Hg exposure.

Spline regression models showed a nonlinear dose–response relationship for log-transformed hair and total mercury (Figure 1). To examine mercury as a continuous variable, we fit adjusted piecewise logistic regression models, with cut points based on visual inspection of the spline graphs. The dose–response relationship for both hair and total blood mercury increased in a statistically significant fashion within the lower ranges of mercury exposure (through –1 ppm log hair mercury and 0 μg/L log total blood mercury) and then plateaued.

**Figure 2:**
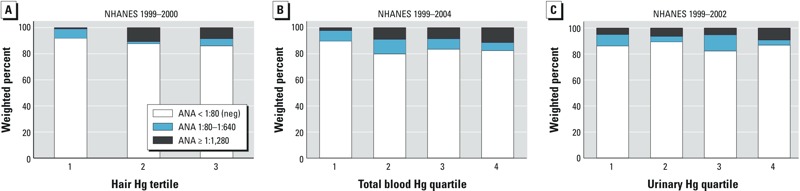
Weighted proportions of antinuclear antibody (ANA) positivity and titer categories according to mercury (Hg) exposure quantiles. (*A*) Hair Hg (1999–2000; *n *= 452). (*B*) Total blood Hg (1999–2004; *n *= 1,352). (*C*) Urinary Hg (1999–2002; *n *= 804).

We also evaluated strength of ANA titer as an outcome. For both hair and total blood mercury, compared with the lowest mercury quantile, the upper quantiles contained a substantially higher proportion of individuals with high-titer ANA (≥ 1:1,280; Figure 2). Consistent with the logistic regression models, results from multinomial logistic regression (see Supplemental Material, Table S2) demonstrated a significant association between hair and total blood mercury, but not urinary mercury, and ANA positivity (data for urinary mercury not shown). Further, magnitudes of association were strongest for high-titer ANA (≥ 1:1,280), where adjusted ORs were > 10 for hair mercury and > 4 for total blood mercury.

## Discussion

In this population-based study, we found that mercury exposure was associated with increased risk of high-titer ANA positivity among reproductive-age females in the general U.S. population. Specifically, this association appears to be driven by organic (methyl) mercury, the predominant species in hair and total blood. Notably, a dose–response relationship was observed for low methylmercury exposure levels (< 0.37 ppm hair mercury; < 1 μg/L total blood mercury), in the range generally considered safe for women of childbearing potential by regulatory agencies (Mergler et al. 2007). The predominant nuclear staining pattern of speckled found in our population is a marker of autoimmunity with a wide variety of clinical associations, including SLE, mixed connective tissue disease, Sjögren’s syndrome, and idiopathic inflammatory myopathies (Morehead 2008). The methylmercury association was robust across models, whereas other suspected risk factors in the multivariable models, including age and smoking, were not associated with ANA risk.

Our findings are compatible with murine data demonstrating development of autoimmunity in response to methylmercury exposure in genetically susceptible strains (Häggqvist et al. 2005; Hultman and Hansson-Georgiadis 1999). Results from human studies have been inconsistent regarding the relationship between organic mercury and autoimmunity. An ecologic study of two Brazilian riverine communities with high fish consumption found a suggestion of higher ANA prevalence in the community with higher average hair mercury levels (8 ppm vs. 6.4 ppm) (Silva et al. 2004). A further study in a riverine Brazilian community failed to detect an association (Alves et al. 2006); the mean hair mercury level in that study was 34.5 ppm, thus it is possible that a dose effect between methylmercury and ANA positivity could have been obscured if, as our data suggest, the response plateaued at a low exposure threshold. A study of females 12–85 years of age from one cycle of NHANES (2003–2004) failed to detect a significant association between total blood mercury and ANA positivity, although the nonlinear nature of association that we observed was not addressed in their analyses (Gallagher et al. 2013). Further, they did not report the titer for defining ANA positivity, and in their smaller sample (632 compared with 1,352 in our blood mercury analyses) statistical power may have been inadequate to detect an association.

Our study focused on females 16–49 years of age. It is well recognized that females have a higher risk of autoimmune diseases (Cooper et al. 2009; Somers et al. 2007, 2013, 2014), and that risk among females may also correlate with reproductive stage. Moreover, estrogenic hormones may promote autoimmunity (Somers and Richardson 2014). Mercury metabolism may also contrast between sexes, and differences in mercury excretion and distribution have been observed between sexes in mouse models (Hirayama and Yasutake 1986; Hirayama et al. 1987), as well as immunotoxic effects at lower internal doses in females (Nielsen and Hultman 2002).

Oxidative stress has been shown to contribute to the induction of autoimmune phenotypes in animal models, such as through epigenetic mechanisms converting normal helper T cells to autoreactive lymphocytes sufficient to cause lupus in the absence of added antigen (Somers and Richardson 2014). Mercury induces oxidative stress through sulfydryl reactivity and depletion of cellular antioxidants (Ercal et al. 2001). In human T cells treated with methylmercury, reductions in intracellular glutathione (GSH) concentration, glutathione *S*-transferase activity, and mitochondrial transmembrane potential have been observed, followed by generation of reactive oxygen species; intracellular GSH depletion has further been linked to susceptibility of T cells to undergo methylmercury-induced apoptosis (Shenker et al. 1999).

It is unclear why we did not find evidence linking inorganic mercury to ANAs because inorganic mercury has been more thoroughly linked to autoimmunity in animal models (Vas and Monestier 2008) and industrially exposed human populations (Cooper et al. 2004; Gardner et al. 2010; Silva et al. 2004). However, the higher doses in such studies limit their generalizability. Indeed, median urinary mercury levels were > 3.7 μg/L in a pair of studies in a Brazilian gold-mining population (Gardner et al. 2010; Silva et al. 2004) (compared with our median of 0.64 μg/L). Another distinction is that these studies used an ANA titer of ≥ 1:10 as detectable as well as a restricted dilution range (to 1:320); a more robust approach would be to employ higher titration levels to better assess strength of ANA positivity. Mechanisms of and degree of immunotoxicity may differ according to level of inorganic mercury. For instance, mercuric chloride at high concentrations (40 μM) has been associated with nonapoptotic cell death, rapid cellular permeabilization (Pollard et al. 1997), and modification of the nucleolar antigen fibrillarin from a 34-kDa non-disulfide–bonded form to a 32-kDa disulfide–bonded form. It is conceivable that structurally altered fibrillarin would be more immunogenic than its native form by unveiling of cryptic epitope(s), and together with cellular necrosis and permeabilization, could be more readily accessible to the immune system. At lower concentrations, fibrillarin migrated at both 32-kDa and the predominant 34-kDa forms, and greater cellular viability was maintained (Pollard et al. 1997). In contrast to inorganic species, organic mercury is lipophilic and more readily crosses cellular membranes, but it may demethylate intracellularly to inorganic mercury (Clarkson and Magos 2006), which may ultimately be more immunotoxic. It is plausible that subcytotoxic levels of organic mercury, such as those in our study, over long periods might yield higher intracellular doses of inorganic mercury and more efficient access to the nuclear environment than would occur with direct exposure to similarly low levels of inorganic mercury.

In our study, we found that speckled patterns predominated (96% of ANA positives). A variety of speckled ANA patterns can be seen by indirect immunofluorescence. Antigen specificities include U1-SnRNP (small nuclear ribonucleoproteins), Sm (Smith), U2-snRNP, U4/U6-snRNP, SSA/Ro, SSB/La, and other less common antigens (Bradwell et al. 2003). In contrast, the nucleolar pattern has primarily been reported in association with inorganic and methylmercury, with specificity of autoantibody formation to fibrillarin/U3RNP demonstrated in murine models in response to mercuric chloride (Hultman et al. 1989; Reuter et al. 1989). A proposed mechanism is that inorganic mercury cross-links with free sulfhydryls on two cysteines of fibrillarin, resulting in physiochemical protein modification (Pollard et al. 1997). Although anti-fibrillarin antibody formation is best recognized in response to inorganic mercury, anti-chromatin and anti-histone antibody formation have also been demonstrated (Hultman et al. 1996). For all three types of autoantibodies, the response varies according to mouse strain, underscoring the relevance of genetic susceptibility. Hultman et al. (1996) demonstrated that antibodies to fibrillarin and chromatin tended to persist several months following cessation of mercuric chloride treatment, whereas anti-histone antibodies resolved more rapidly. We found only 14 cases with the nucleolar pattern, none of which demonstrated anti-fibrillarin antigenicity upon immunoprecipitation. Only 3 cases had a nuclear homogeneous pattern, which would be compatible with histone or chromatin antigens. In humans, the nucleolar pattern, and particularly anti-fibrillarin antibodies, are associated with scleroderma, especially among blacks and males (Arnett et al. 1996). The rarity of scleroderma (prevalence ~ 27.6/100,000 adults) (Mayes et al. 2003) and its associated autoantibodies make it unlikely that our study would have adequate power to detect an association with these specific autoantibodies. However, it is difficult to explain why the speckled pattern was prominent in our study but not in the animal literature or human occupational studies. Whether organic mercury preferentially targets different nuclear antigens than does inorganic mercury, or whether an alternate biologic pathway is relevant to low compared with high doses of either species, remains to be elucidated.

Fish consumption is an exposure route common to both methylmercury and essential nutrients that may have beneficial impact on the immune system. Both organic and inorganic mercury are suggested to increase the production of prostaglandin E2 and phospholipase A2 (Mazerik et al. 2007), leading to release of arachidonic acid. Omega-3 fatty acids are an alternative substrate to arachidonic acid for cyclooxygenase and lipoxygenase enzymes, and they induce a series of anti-inflammatory eicosanoids (Simopoulos 2002). Thus, we incorporated omega-3 fatty acids into our modeling because of the potential for negative confounding (Choi et al. 2008). There was indeed a modest increase in the magnitudes of association for hair mercury when adjusting for omega-3 intake. PCBs are persistent toxicants with suggested immune effects (Gallagher et al. 2013; Heilmann et al. 2010) and fish consumption as an exposure route. The hair and total blood mercury associations were not appreciably altered with inclusion of PCBs in the models.

Limitations of our study include its cross-sectional nature, precluding the ability to determine the pattern and chronicity of mercury exposure, and persistence of ANA positivity or future risk of overt disease. However, the study of risk factors for preclinical disease is an important tool for dissecting the etiology of complex diseases with long latencies (Cooper 2009). Further, certain combinations of autoimmune diseases tend to co-occur within individuals and families (Cooper et al. 2009; Somers et al. 2006, 2009) to an extent inadequately explained by genetic background. The identification of shared environmental factors for immune dysregulation relevant to a variety of autoimmune phenotypes is an important goal (Dietert et al. 2010). The nonspecific nature of the ANA patterns documented here supports the premise of organic mercury as a risk factor for multiple autoimmune conditions. Future research is necessary to evaluate whether our study findings extend to other populations, including males and persons outside of the 16- to 49-year-age range.

## Conclusions

We provide evidence for the first time, to our knowledge, that low levels of methylmercury exposure are linked to subclinical autoimmunity among females of reproductive age in the general population. Because autoantibody development is a marker of immune dysregulation and may predate clinical autoimmune diagnoses by several years, the prospect that organic mercury acts as an early but potentially modifiable trigger relevant to a spectrum of autoimmune conditions warrants more intense investigation.

## Supplemental Material

(244 KB) PDF
