# Supplementary material for: Mercury Exposure and Antinuclear Antibodies among Females of Reproductive Age in the United States: NHANES
Source: Environ Health Perspect. 2015 Feb 10;123(8):792–8. doi: 10.1289/ehp.1408751 (PMC4529012; doi:10.1289/ehp.1408751)
Supplement: (244 KB) PDF [file ehp.1408751.s001.508.pdf]

Environmental Health Perspectives doi:10.1289/ehp.1408751

Supplemental Material

Mercury Exposure and Antinuclear Antibodies among Females of Reproductive Age in the United States: NHANES

Emily C. Somers, Martha A. Ganser, Jeffrey S. Warren, Niladri Basu, Lu Wang, Suzanna M. Zick, and Sung Kyun Park

Table S1. Association between mercury exposure and ANA positivity among females 16-49 years in the general US population (NHANES), with adjustment for polychlorinated biphenyls (PCBs).

Mercury exposure	ANA positive n (%)[a]	Crude Model[b] OR (95% CI)	Coplanar PCBs[c,d] OR (95% CI)	Prevalent PCBs[c,e] OR (95% CI)
**Hair mercury (ppm)[f]**				
Tertile 1 (<0.11)	13 (8)	1.00 (referent)	1.00 (referent)	1.00 (referent)
Tertile 2 (0.11-0.27)	22 (13)	1.61 (0.27, 9.64)	2.71 (0.54, 13.55)	2.65 (0.57, 12.30)
Tertile 3 (0.271-5.96)	20 (15)	1.96 (0.57, 6.77)	4.44 (1.60, 12.28)	4.03 (1.65, 9.84)
**Total blood mercury (μg/L)[g]**				
Quartile 1 (<0.4)	27 (9)	1.00 (referent)	1.00 (referent)	1.00 (referent)
Quartile 2 (0.4-0.8)	67 (21)	2.52 (1.16, 5.47)	2.70 (1.29, 5.67)	2.30 (1.11, 4.78)
Quartile 3 (0.9-1.5)	50 (17)	2.04 (0.95, 4.39)	2.43 (1.12, 5.27)	2.07 (0.97, 4.42)
Quartile 4 (1.6-32.8)	61 (18)	2.10 (0.98, 4.50)	2.71 (1.25, 5.86)	2.33 (1.09, 4.98)

Abbreviations: ANA, antinuclear antibody; PCBs, polychlorinated biphenyls; Hg, mercury; NHANES, National Health and Nutrition Examination Survey; OR, odds ratio.

[a]Weighted percent. [b]Models include subset with available PCB data (samples size reduced by ~6% compared to main analyses). [c]Adjusted for age, race/ethnicity, education, serum cotinine, selenium, omega-3 fatty acids, and an indicator for NHANES cycle for models combining data across cycles (1999-2004). [d]Coplanar (dioxin-like) PCB congeners included PCBs (81, 105, 118, 126, 156, 157, 167, 169); a summary measure was calculated as the sum of the products of the concentration of each serum lipid-adjusted congener and its corresponding toxic equivalency factor (TEF), which was then log-transformed. [e]The Prevalent PCB measure is the log-transformed sum of the lipid-adjusted values for the four most prevalent PCB congeners (PCBs 118, 138, 153, 180), three of which are non-coplanar and without defined TEFs to take into account. [f]NHANES 1999-2000, 1 cycle (n=424). [g]NHANES 1999-2004, 3 cycles (n=1281).

Table S2. Association between mercury exposure and ANA titer strength, among females 16-49 years in the general US population (NHANES), based on multinomial logistic regression modeling.

Mercury exposure	ANA positive n (%)[a]	Crude Model OR (95% CI)	Model A[b] OR (95% CI)	Model B[c] OR (95% CI)	Model C[d] OR (95% CI)
**Hair mercury (ppm)[e]**					
**ANA negative (n=396)**	NA	NA	NA	NA	NA
**ANA 1:80-1:640 (n=32)**					
Tertile 1 (<0.11)	11 (7)	1.00 (referent)	1.00 (referent)	1.00 (referent)	1.00 (referent)
Tertile 2 (0.11-0.27)	9 (2)	0.23 (0.03, 2.04)	0.23 (0.04, 1.32)	0.24 (0.04, 1.38)	0.20 (0.02, 1.64)
Tertile 3 (0.271-5.96)	12 (6)	0.82 (0.40, 1.69)	2.93 (1.22, 7.06)	2.98 (1.29, 6.90)	2.11 (0.73, 6.15)
**ANA ≥1:1280 (n=24)**					
Tertile 1 (<0.11)	3 (1)	1.00 (referent)	1.00 (referent)	1.00 (referent)	1.00 (referent)
Tertile 2 (0.11-0.27)	13 (11)	11.81 (0.98, 142.86)	13.74 (1.09, 172.78)	16.69 (1.63, 171.23)	14.29 (1.02, 200.25)
Tertile 3 (0.271-5.96)	8 (8)	9.47 (1.43, 62.60)	11.14 (1.53, 81.36)	11.41 (1.60, 81.23)	12.57 (1.36, 116.44)
**Total blood mercury (μg/L)[f]**					
**ANA negative (n=1139)**	NA	NA	NA	NA	NA
**ANA 1:80-1:640 (n=109)**					
Quartile 1 (<0.4)	20 (8)	1.00 (referent)	1.00 (referent)	1.00 (referent)	1.00 (referent)
Quartile 2 (0.4-0.8)	40 (11)	1.55 (0.66, 3.62)	1.61 (0.64, 4.10)	1.62 (0.64, 4.09)	1.63 (0.59, 4.55)
Quartile 3 (0.9-1.5)	23 (8)	1.06 (0.46, 2.42)	1.37 (0.56, 3.38)	1.39 (0.57, 3.36)	1.27 (0.45, 3.55)
Quartile 4 (1.6-32.8)	26 (6)	0.82 (0.40, 1.70)	1.10 (0.45, 2.70)	1.11 (0.46, 2.69)	1.09 (0.35, 3.41)
**ANA ≥1:1280 (n=104)**					
Quartile 1 (<0.4)	10 (2)	1.00 (referent)	1.00 (referent)	1.00 (referent)	1.00 (referent)
Quartile 2 (0.4-0.8)	31 (9)	4.70 (1.30, 16.98)	4.23 (1.10, 16.24)	4.20 (1.09, 16.24)	4.42 (1.15, 16.99)
Quartile 3 (0.9-1.5)	28 (8)	4.35 (1.27, 14.82)	4.21 (1.18, 15.09)	4.13 (1.15, 14.80)	4.57 (1.26, 16.60)
Quartile 4 (1.6-32.8)	35 (11)	5.91 (1.70, 20.56)	6.04 (1.59, 22.86)	5.93 (1.57, 22.47)	6.17 (1.61, 23.70)

Abbreviations: ANA, antinuclear antibody; Hg, mercury; NHANES, National Health and Nutrition Examination Survey; OR, odds ratio

[a]Weighted percent. [b]Adjusted for age, race/ethnicity, education, serum cotinine, selenium, and an indicator for NHANES cycle for multi-cycle models. [c]Model A + further adjusted for omega-3 fatty acids. [d]Model A + further adjusted for total seafood intake. [e]NHANES 1999-2000, 1 cycle (n=452). [f]NHANES 1999-2004, 3 cycles (n=1352).
